# Effect of Glyoxal on Plasma Membrane and Cytosolic Proteins of Erythrocytes

**DOI:** 10.3390/ijms26094328

**Published:** 2025-05-02

**Authors:** Michal Kopera, Malgorzata Adamkiewicz, Anna Pieniazek

**Affiliations:** 1 Department of Oncobiology and Epigenetics, Faculty of Biology and Environmental Protection, University of Lodz, Pomorska 141/143 Str., 90-236 Lodz, Poland; michal.kopera@edu.uni.lodz.pl (M.K.); malgorzata.adamkiewicz@edu.uni.lodz.pl (M.A.); 2 Doctoral School of Exact and Natural Sciences, University of Lodz, 21/23 Jana Matejki Str., 90-236 Lodz, Poland

**Keywords:** glyoxal, erythrocyte, hemoglobin, antioxidant enzymes, glutathione

## Abstract

Glyoxal (GO) is a reactive dicarbonyl derived endogenously from sugars and other metabolic reactions within cells. Numerous exogenous sources of this compound include tobacco smoking, air pollution, and food processing. GO is toxic to cells mainly due to its high levels and reactivity towards proteins, lipids, and nucleic acids. We speculate that glyoxal could be involved in erythrocyte protein damage and lead to cell dysfunction. The osmotic fragility and level of amino and carbonyl groups of membrane proteins of erythrocytes incubated for 24 h with GO were identified. The amount of thiol, amino, and carbonyl groups was also measured in hemolysate proteins after erythrocyte treatment with GO. In hemolysate, the level of glutathione, non-enzymatic antioxidant capacity (NEAC), TBARS, and activity of antioxidant enzymes was also determined. The study’s results indicated that GO increases erythrocyte osmotic sensitivity, alters the levels of glutathione and free functional groups in hemolysate proteins, and modifies the activity of antioxidant enzymes. Our findings indicate that GO is a highly toxic compound to human erythrocytes. Glyoxal at concentrations above 5 mM can cause functional changes in erythrocyte proteins and disrupt the oxidoreductive balance in cells.

## 1. Introduction

Glyoxal (GO) is a highly reactive dicarbonyl compound that can interact with various cell biomolecules [1]. Endogenous sources of GO include various metabolic and oxidative reactions in cells. These processes include, among others, the breakdown of sugars, lipids, and proteins, resulting in the formation of GO. Glyoxal can be formed from glucose via the Maillard reaction or the breakdown of glycolytic intermediates [2]. Other sources of endogenous GO may be lipid peroxidation, amino acid, and glycated protein degradation, as well as nucleotide degradation [3]. GO can also enter cells through environmental exposures, including tobacco smoking, air pollution, and specific food processing methods that produce carbonyl compounds [2].

The high level of GO can have several detrimental effects on cells, largely due to its reactivity and ability to form adducts with proteins, lipids, and nucleic acids [4]. GO is highly reactive and can covalently bind to the amino groups of proteins, leading to the formation of advanced glycation end products (AGEs). AGEs form gradually through the Maillard reaction over weeks, but most AGEs in living organisms are produced rapidly via dicarbonyl compounds like GO reacting with proteins [5]. These protein modifications can impair protein structure and function. It is also suggested that elevated GO concentrations in cells cause an increase in the production of reactive oxygen species (ROS) [6,7,8]. In cells undergoing oxidative stress, ROS can interact with cellular components such as lipids, proteins, sugars, and DNA, contributing to the formation of GO. This oxidative stress can further exacerbate cellular dysfunction and contribute to inflammatory responses. There are reports in the literature indicating that GO interacts with nucleic acids. Such interactions have been shown to cause DNA damage, mutations, and impaired gene expression [8,9]. Many studies indicate a decrease in glutathione levels in cells exposed to GO, which further impairs the antioxidant system in cells [1,10]. Interestingly, Nomi et al. suggest that reduced glutathione reacted with GO at the α-NH_2_ group of the glutamate residue, but not at the SH group of the cysteine residue [11].

High levels of glyoxal are linked to various diseases due to its toxic effects on cellular components. The link between elevated GO levels and disease primarily arises from its role in forming AGEs and its capacity to induce oxidative stress, inflammation, and cellular dysfunction. Diabetes, particularly when blood sugar levels are poorly controlled, can lead to serious complications. High glucose levels result in increased production of GO, which contributes to the formation of AGEs. These AGEs can accumulate in tissues over time, causing long-term damage to blood vessels, nerves, kidneys, and the eyes. Studies conducted by Dhananjaya et al. have shown that the level of GO in the serum of diabetic patients is about twice as high as in healthy individuals [12]. Similarly, significantly higher levels of this compound were observed in patients with chronic kidney disease [13,14]. GO has been implicated in the progression of neurodegenerative conditions, such as Alzheimer’s disease [1]. In neurodegenerative diseases, GO-induced protein glycation leads to the formation of protein aggregates that disrupt normal cellular function. 

Due to the reactivity and versatility of GO’s actions and the influence of this compound on the progression of many diseases, we accepted the challenge of assessing the effect of this compound on erythrocytes in vitro. The primary focus was on the modification of membrane and cytoplasmic proteins due to increased GO concentration and the role of this compound in inducing oxidative stress in erythrocytes.

## 2. Results

**Figure 1:**
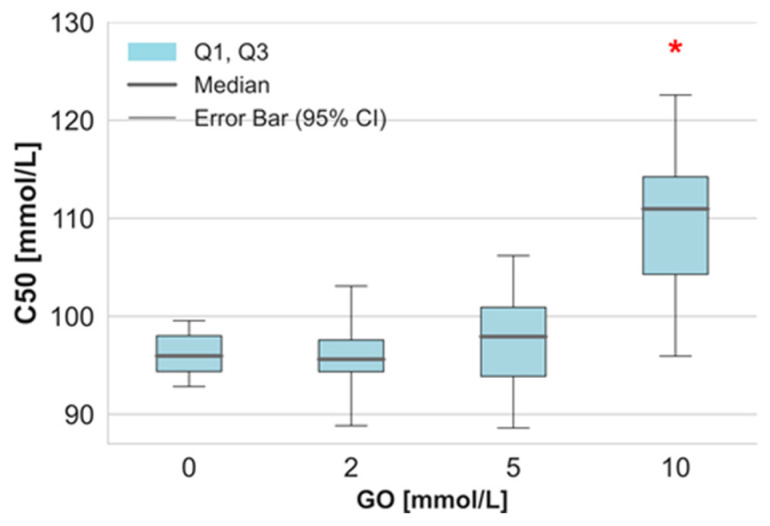
The osmotic fragility of the RBCs after incubation with GO (C_50_—NaCl concentration at which 50% of RBCs undergo hemolysis). Data were presented as median with a boxplot bounded by quartiles, n = 10, * *p* < 0.05—GO (10 mM) versus control.

The obtained results demonstrate how GO affects oxidative stress markers, protein modifications, and antioxidant defenses of human RBCs. In this study, RBCs were incubated with GO at final concentrations of 2 mM, 5 mM, and 10 mM for 24 h at 37 °C. After incubation, we assessed the osmotic fragility. The results indicated that RBCs exposed to the highest GO (10 mM) exhibited significantly increased sensitivity to hemolysis compared to the control group (Figure 1). In contrast, the lower GO concentrations (2 mM and 5 mM) did not notably affect the osmotic resistance of erythrocytes.

**Figure 2:**
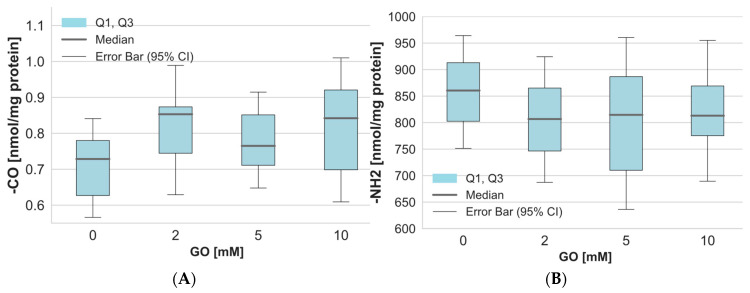
The level of (**A**) carbonyl and (**B**) amino groups in erythrocyte plasma membrane proteins after incubation of whole erythrocytes with GO. Data were presented as median with a boxplot bounded by quartiles, n = 10.

The levels of carbonyl groups, a marker of protein oxidation, were assessed in both membrane and hemolysate fractions. The obtained results indicate a slight increase in carbonyl content in plasma membrane proteins upon GO treatment (Figure 2A). The measurement of amino groups in plasma membrane proteins revealed a slight decrease in their levels following GO exposure (Figure 2B). This decline suggests that GO reacts with free amino groups, leading to protein cross-linking and advanced glycation end-product (AGE) formation. However, those changes that occurred were not statistically significant.

**Figure 3:**
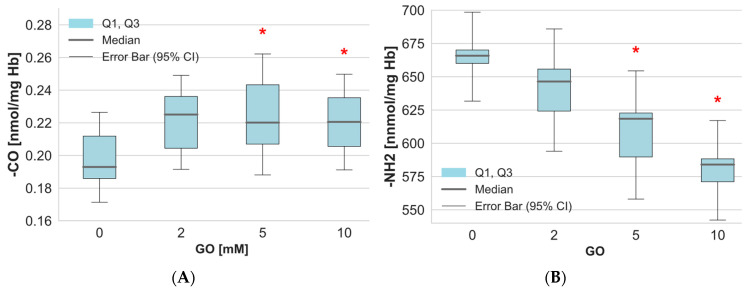
The level of (**A**) carbonyl and (**B**) amino groups in hemolysate proteins of erythrocytes after incubation of whole erythrocytes with GO. Data were presented as median with a boxplot bounded by quartiles, n = 10 for carbonyl groups, and n = 9 for amino groups. * *p* < 0.05—GO (5 mM) vs. control and GO (10 mM) vs. control.

On the other hand, the determination of the level of carbonyl and amino groups in the cytoplasmic proteins of erythrocytes incubated with GO showed statistically significant changes compared to the control values (Figure 3A). The measurement of amino groups in hemolysate proteins revealed a noticeable decrease in their levels following RBCs GO exposure (Figure 3B). It is interesting to note that statistically significant changes were observed in the levels of carbonyl and amino groups in hemolysate proteins when RBCs were incubated with GO at concentrations of 5 mM and above.

**Figure 4:**
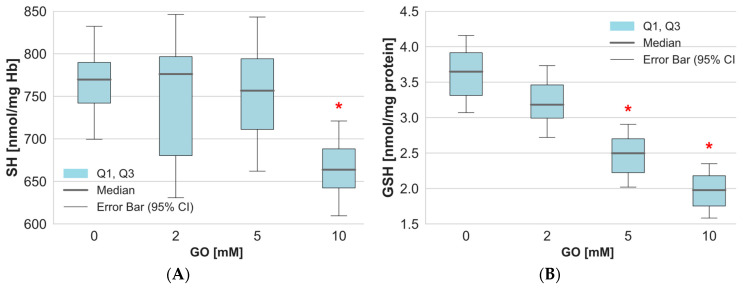
The level of (**A**) thiol group and (**B**) GSH in hemolysate proteins of erythrocytes after incubation of whole erythrocytes with GO. Data were presented as median with a box plot bounded by quartiles, n = 10. * *p* < 0.05—GO (5 mM) vs. control and GO (10 mM) vs. control.

Under oxidative stress conditions, the modification of protein functional groups is very common. Free thiol groups of proteins are characterized by their high sensitivity to various factors. The study showed a significant decrease in the level of thiol groups in the cytoplasmic proteins of erythrocytes treated with GO at a concentration of 10 mM (Figure 4A). Lower GO concentrations did not result in changes to this parameter in the proteins. Low-molecular-weight thiols, particularly glutathione, are an important component of an efficiently functioning antioxidant system in cells. The results clearly show a significant decrease in glutathione levels in GO-treated RBCs (Figure 4B). It is worth noting that a significant decrease in GSH levels was observed in RBCs at a concentration of 5 mM and above.

**Figure 5:**
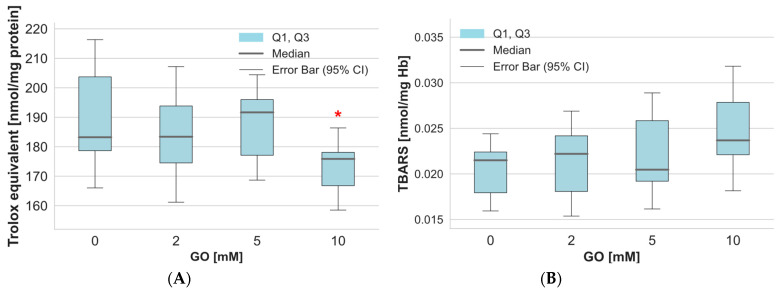
(**A**) The total non-enzymatic antioxidant capacity and (**B**) the concentration of thiobarbituric acid-reactive substances (TBARS) in hemolysate proteins of erythrocytes incubated with GO. Data were presented as median with a boxplot bounded by quartiles, n = 9 for TBARS and n = 10 for NEAC, * *p* < 0.05—GO (10 mM) vs. control.

The consequence of oxidation-reduction disorders in cells can be changes in the efficiency of the total non-enzymatic antioxidant capacity in cells (NEAC), which consists mainly of low-molecular-weight antioxidants. Our results of NEAC studies in erythrocytes treated with GO showed slight changes in this parameter at low toxin concentrations and a significant decrease in RBCs treated with GO at a concentration of 10 mM (Figure 5A). We also checked the levels of thiobarbituric acid-reactive substances in cells treated with GO. The analysis results indicated a minor increase in this parameter due to GO when compared to the control samples (Figure 5B).

**Figure 6:**
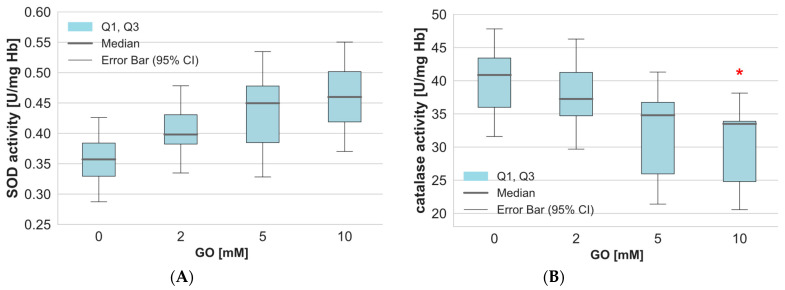
Alterations in the SOD (**A**) and catalase (**B**) activity in hemolysate after incubation of whole erythrocytes with GO. Data are expressed as median with a box plot bounded by quartiles, n = 9. * *p* < 0.05—GO (10 mM) vs. control.

A crucial element in maintaining the oxidation-reduction balance in cells is the enzyme system that converts reactive oxygen species into non-toxic products. In our work, we examined SOD activity in GO-treated RBCs. The results showed no statistically significant changes in superoxide dismutase activity in glyoxal-treated erythrocytes (Figure 6A). On the other hand, catalase activity in RBCs treated with GO at a concentration of 10 mM significantly decreased compared to the values obtained in control cells (Figure 6B). Lower GO concentrations did not cause significant changes in catalase activity in the cells tested.

## 3. Discussion

People are constantly exposed to many toxic substances. However, a well-functioning metabolism enables, on the one hand, the elimination of toxins from the body and, on the other, the development of defense mechanisms that guard against the effects of harmful substances. Data on GO toxicity and potential sources of this compound to which humans are exposed are described in Concise International Chemical Assessment Document 57, World Health Organization (Geneva 2004). In vivo studies have shown that in healthy people, the plasma GO level ranges from approximately 100 nM to 500 nM. In turn, in people with diabetes, the concentrations of this compound in plasma are much higher (up to 1500 nM) [12,15]. To determine the mechanism of action of GO in vitro, other researchers used glyoxal at significantly higher concentrations, reaching up to 10 mM [7,10,11,16,17]. Before starting the study, we also performed a preliminary test of the survival of mononuclear blood cells exposed to different GO concentrations, which showed 40% survival of cells exposed to GO (10–20 mM) for 24 h. Considering the literature data, our research, and the observation that erythrocytes are less sensitive to unfavorable factors than mononuclear cells, we selected three concentrations of GO (2, 5, and 10 mM) for this study. The study involved exposing isolated erythrocytes to glyoxal at three different concentrations for 24 h at a temperature of 37 °C.

Considering that GO is classified as a protein-binding uremic toxin [14,18], studies were conducted on the modification of functional groups of erythrocyte membrane proteins exposed to this compound. Our studies have shown a slight increase in the level of carbonyl groups and a decrease in the level of amino groups in plasma membrane proteins. This finding appears to align with the known mechanisms of action for GO (protein carbonylation and GO reactions with amino groups). However, the obtained results do not allow for drawing firm conclusions for plasma membrane proteins. In previous studies on several proteins (alpha lactalbumin, myoglobin, lysozyme, carbonic anhydrase), it was shown that, under the influence of GO, the level of carbonyl groups increased in these proteins. The authors of these studies also observed changes in the hydrodynamic diameter and in the secondary structures of the tested proteins, which suggest the interaction of the protein with GO [19]. This may suggest that, in our study, erythrocyte plasma membrane proteins are not the primary targets of the reaction with GO. The reaction of the amino groups of proteins with GO leads to the formation of the Schiff base (Protein−N=CH−CHO). The product of this reaction is quite unstable and can undergo further reactions to form more stable advanced glycation end products (AGEs). It appears that the formation of the Schiff base in the reaction of GO with the amino groups of proteins may be responsible for the increasing amount of carbonyl groups in proteins. In parallel with the statistically significant decrease in the level of amino groups in the hemolysate proteins, a significant increase in the level of carbonyl groups was also observed. In turn, for erythrocyte membrane proteins, the changes in both parameters were insignificant. It has been previously described that glyoxal, which has two carbonyl reactive groups, easily participates in glycation reactions that lead to the development of carbonyl stress [20].

Currently, there are no specific details about how GO is transported into cells. However, research has shown that GO can cross cell membranes through both passive diffusion and active transport mechanisms [21]. Although the authors of the study suggest that the transport of GO into cells is limited, the intracellular effects of GO may be enhanced because this compound is also produced endogenously in cells. The study we presented here showed significantly higher osmotic sensitivity of GO-treated erythrocytes. This effect might result from GO’s interaction with membrane lipids or its passive transport into the cells. Confirmation of the changes occurring in the membranes of GO-treated erythrocytes can also be provided by studies showing that GO significantly reduced the deformability of these cells [17]. The authors of the paper state that the primary cause of changes in erythrocyte deformability is the Maillard reaction between erythrocyte proteins and carbonyl compounds. Morphological changes in bovine pulmonary artery endothelial cells (BPAECs) under the influence of glyoxal were also observed by Gurney et al. [22]. In addition, the authors of this study showed a significant decrease in cell survival and an increase in the level of reactive oxygen species in BPAECs after 24-h incubation with GO at concentrations 10 times lower than in our study.

While erythrocyte membrane proteins showed insignificant changes in functional groups, in the case of hemolysate proteins, GO caused a significant increase in carbonyl groups and a significant decrease in thiol and amino groups. The research on isolated hemoglobin’s interaction with GO showed various modifications to this protein [23]. The changes in hemoglobin caused by GO primarily affect its secondary structure. As is known, changes in the composition of free functional groups in proteins significantly affect the formation of their secondary structures. Other studies of the effect of GO on the level of carbonyl groups in hemoglobin have shown a decrease in this parameter [17]. These results are contrary to those obtained in our study. However, the protocol for conducting the experiments was quite different. Glycation modifications change hemoglobin’s (Hb) structure and function, affecting its oxygen-binding ability and signaling through Band3. Both Band3 and GAPDH can be glycated, playing a crucial role in cellular energy regulation and the physiological state of erythrocytes [24]. Such changes in erythrocyte membrane proteins may also be responsible for the loosening of the membrane structure and greater sensitivity to hemolysis.

Another important phenomenon observed in GO-treated hemoglobin was significant iron release from heme [23]. Under certain conditions, iron released from hemoglobin can react with hydrogen peroxide to generate reactive hydroxyl radicals through the Fenton reaction, which is known to cause oxidative stress in cells. Generally, many times, it has already been shown that the cytotoxic mechanism of GO involves oxidative stress [6,7,10], that reactive oxygen species (ROS) can be generated by AGEs [25]. Studies in hepatocytes have shown that 5 mM of GO induce a significant increase in cellular levels of ROS [10]. Similar results for ROS levels (increase relative to control) in GO-treated human retinal pigment epithelial (ARPE-19) cells were obtained by Roehlecke et al. [7]. Shangari O’Brien also showed a decrease in the concentration of reduced glutathione in GO-treated cells, which is consistent with our results obtained in erythrocytes [10]. The authors of this study also observed an increase in levels of the oxidized form of glutathione and a decrease in glutathione reductase activity in cells exposed to GO, indicating increased oxidative processes in the cells [10]. A 55% decrease in intracellular GSH was observed in human aortic endothelial cells (HAECs) incubated with GO, relative to the control [1]. Since decreased glutathione levels are observed in GO-treated cells, clarifying the interaction mechanism between the two molecules is crucial. Research by Nomi and colleagues has shown that the cysteine thiol group in GSH is not the primary reaction target for GO [11]. The authors of this paper suggest that GO reacts with the N-terminal amine group of GSH. This reaction ultimately results in two products that are in equilibrium with each other: N-Glycoloyl-glutamylcysteinylglycine and N-[3-(2,5-Dioxomorpholin-3-yl)-propanoyl]cysteinylglycine [11]. The biological role of the resulting products is not known. It is not excluded that GO may similarly react with the free amino groups of proteins. The statistically significant decrease in the level of amino groups in the cytoplasmic proteins of erythrocytes incubated with GO that we observed may be due to such reactions. The reduction in thiol groups of hemolysate proteins is mainly due to oxidation processes caused by reactive oxygen species. An increase in the level of carbonyl groups and a decrease in the level of thiol groups of proteins under oxidative stress conditions have previously been observed in both erythrocytes and cells containing a cell nucleus [26,27]. A paper on the mechanism of action of another dialdehyde (methylglyoxal) suggests that the carbonyl stress and oxidative stress induced by this toxin may lead erythrocytes into the pathway of eryptosis [24].

Oxidative stress results from an imbalance between the level of production of reactive oxygen species and the efficiency of the antioxidant system (enzymatic and non-enzymatic) [28]. The non-enzymatic system includes the effectiveness of low-molecular-weight (LMW) antioxidants, both water- and fat-soluble. The results indicated a reduction in antioxidant potential in GO-treated erythrocytes. This outcome is linked to a decrease in reduced glutathione, a low-molecular-weight antioxidant. The enzymatic antioxidant system involves the action of several enzymes responsible for the breakdown of reactive oxygen species. Our study demonstrates a decrease in catalase activity in erythrocytes incubated with 10 mM of GO. Previous studies of antioxidant enzymes activity in erythrocytes incubated with GO revealed no changes in catalase activity but a significant decrease in SOD activity [29]. Jabeen et al. showed that isolated superoxide dismutase treated with GO at a concentration of 10 mM loses 95% of its activity [30]. It has been suggested that GO interacts with SOD, creating advanced glycation end-products (AGEs) that change its conformation and reduce its ability to bind essential metal cofactors like copper and zinc [31]. In contrast, studies of normal adult human skin fibroblasts (ASF-2) exposed to GO (1 mM) did not reveal any significant differences in the activity of either SOD or catalase [16]. Another study conducted on aging retinal pigment epithelial (ARPE-19) cells showed that GO increased protein expression of HO-1, osteopontin, Hsp27, Mn SOD), Cu/Zn SOD, and cathepsin D relative to the untreated control [7].

The studies presented in this paper were conducted in vitro, where only isolated erythrocytes in Ringer’s buffer and the toxin (GO) were in the test system. Such a research model allows the direction of the toxin-induced changes in cells to be determined but excludes the effect of GO on the whole blood tissue. It appears that treatment with whole blood GO could reduce the observed effects. Due to the high reactivity of GO towards proteins, in whole blood, it is the plasma proteins (mainly albumin) that would be most likely to bind to this toxin.

## 4. Materials and Methods

### 4.1. Chemicals 

The following reagents were obtained from Sigma Chemical Co. (St. Louis, MO, USA): 5,5′-dithiobis(2-nitrobenzoic acid) (DTNB), 4,4′-dithiodipyridine, 2,4,6-trinitrobenzene sulfonic acid (TNBS), 2,4-dinitrophenylhydrazine (DNPH), and 2,4,6-tripyridyl-S-triazine (TPTZ), and glyoxal (GO). Unless stated otherwise, all other chemicals were sourced from POCH S.A. (Gliwice, Poland). 

### 4.2. Sample Preparation 

Erythrocytes (RBCs) were isolated from human blood buffy coat, acquired from the Blood Bank in Lodz, Poland. The isolation process involved centrifugation and washing three times with phosphate-buffered saline (PBS, 10 mM, pH 7.4). The erythrocytes were diluted in Ringer’s buffer to achieve 50% hematocrit and incubated with GO (final concentration of GO 2, 5, and 10 mM) for 24 h at 37 °C. Control samples contained PBS instead of GO. After the end of incubation, the RBCs were washed with PBS. 

Plasma membranes were extracted using Dodge et al.’s method, and membrane protein concentrations were measured using the Folin–Ciocalteu assay, with absorbance at 750 nm [32,33]. 

Hemolysate was prepared by mixing RBCs with cold water (1:1.5 ratio), shaking, and centrifugation to separate membranes. Hemoglobin concentration in the hemolysate was determined spectrophotometrically as cyanmethemoglobin using Drabkin’s reagent, measured at 540 nm [34]. 

### 4.3. Measurement of Osmotic Fragility 

The osmotic fragility of RBCs was assessed following Morimoto’s method [35]. Cells were incubated (0.5 h) in NaCl solutions of varying concentrations (0–155 mM), and hemolysis was determined by measuring the absorbance of the supernatant at 540 nm. The NaCl concentration causing 50% hemolysis (C50) was calculated from the results. 

### 4.4. Measurement of Catalase (CAT) Activity 

Catalase activity in hemolysate was determined according to the method described by Aebi [36] by monitoring the breakdown of hydrogen peroxide. The decrease in absorbance at 240 nm was recorded, and activity was expressed in units (U) per milligram Hb per minute, where 1 U of catalase corresponds to the decomposition of 1 μmol of hydrogen peroxide per minute. 

### 4.5. Measurement of Superoxide Dismutase (SOD) Activity 

SOD activity in hemolysate was analyzed based on its ability to inhibit the auto-oxidation of adrenaline to adrenochrome in alkaline conditions (pH 10.2) [37]. The increase in absorbance at 480 nm was monitored, and the activity was expressed as units (U) per milligram of Hb per minute. 

### 4.6. Determination of Thiobarbituric Acid-Reactive Substances (TBARS) 

Lipid peroxidation in hemolysate was analyzed by measuring TBARS [38]. Under acidic conditions, lipid oxidation products react with thiobarbituric acid to form colored complexes, detectable at 535 nm. The TBARS concentration was calculated using a molar extinction coefficient of (156 mmol^−1^·cm^−1^) and expressed as nanomoles per milligram of Hb.

### 4.7. Determination of Total Non-Enzymatic Antioxidant Capacity (NEAC) 

The NEAC of hemolysate was determined by using 2,2-diphenyl-1-picrylhydrazyl (DPPH), where antioxidants reduce the reagent, decreasing its absorbance at 517 nm [39]. Antioxidant capacities were calibrated against Trolox (0–1 mM) and expressed as nanomoles of Trolox equivalents per milligram of Hb. 

### 4.8. Determination of Free Thiol Group Content 

The levels of free-thiol groups in membrane proteins were analyzed using Ellman’s method [40]. The reaction of Ellman’s reagent (5,5′-dithiobis(2-nitrobenzoic acid), DTNB) with thiol groups forms 2-nitro-5-thiobenzoate (NTB), which exhibits an absorbance at 412 nm.

For hemolysate thiol groups, 4,4′-dithiodipyridine was used as the reagent, producing 2-thiopyridone, which was measured spectrophotometrically at 324 nm [41]. Calibration curves prepared with reduced glutathione (0–1 mM) served to calculate thiol concentrations, expressed as nanomoles per milligram of membrane protein or Hb. 

### 4.9. Determination of Free-Amino Group Content 

The concentration of the free-amino groups in hemolysate and membrane proteins was measured using the method described by Crowell et al [42]. In this approach, 2,4,6-trinitrobenzene sulfonic acid (TNBS) reacts with amino groups to form a chromophore detectable at 335 nm. The amino group concentration was derived from a calibration curve generated with homocysteine solutions (0–250 μM) and reported as nanomoles per milligram of membrane protein or Hb.

### 4.10. Measurement of Carbonyl Group Content 

Carbonyl groups in hemolysate and plasma membrane proteins were measured using the Levine et al. method [43]. This involves the reaction of 2,4-dinitrophenylhydrazine (DNPH) with protein carbonyl groups to form dinitrophenylhydrazones (DNP), which exhibit absorbance at 370 nm. The concentration of carbonyl groups was calculated using a milimolar absorption coefficient of 22 mmol^−1^·cm^−1^ and expressed as nanomoles per milligram of membrane protein or Hb.

### 4.11. Statistical Analysis

Statistical analysis of the data involves checking the normality of the distribution of the parameters tested using the Shapiro–Wilk test. Then, the homogeneity of variances was checked using Levene’s test. The nonparametric Kruskal–Wallis test (also called “one-way ANOVA on ranks”) was used to compare differences between the tested groups. After the Kruskal–Wallis test, multiple comparisons of mean ranks were made for all samples. Statistical significance was accepted at *p* < 0.05 and demonstrated for parameters for which the power of the test was above 0.8. Statistical analysis was performed using Statistica v. 13.3 (StatSoft Polska, Krakow, Poland). The data in the figures were presented as median with a box of values of the first and third quarters. 

## 5. Conclusions

Glyoxal is a highly reactive dicarbonyl compound to which humans are often exposed. Its danger lies in the fact that GO can originate from both endogenous and exogenous sources. However, the molecular mechanisms of GO action on cells are incompletely understood and require further research. Our research shows that this compound affects the osmotic fragility of erythrocytes, potentially shortening their lifespan. Significant alterations occur in the cytoplasm of erythrocytes, including a decrease in glutathione levels, modifications in proteins such as hemoglobin, and changes in the activity of antioxidant enzymes. Research results expand knowledge of GO toxicity and may help develop methods to counter dialdehyde toxicity. Analyzing the results of our own and other researchers’ studies, it seems that the effect of GO depends on both the dose of this toxin and the type of cells.

## Data Availability

No new data were created or analyzed in this study. Data sharing is not applicable to this article.

## Abbreviations

The following abbreviations are used in this manuscript:

AGEs	Advanced glycation end-products
CAT	Catalase
DPPH	2,2-diphenyl-1-picrylhydrazyl
GO	Glyoxal
GSH	Glutathione
NEAC	Non-enzymatic antioxidant capacity
RBCs	Erythrocytes
ROS	Reactive oxygen species
SOD	Superoxide dismutase
TBARS	Thiobarbituric acid reactive substances

## References

[1] Xie M.-Z., Guo C., Dong J.-Q., Zhang J., Sun K.-T., Lu G.-J., Wang L., Bo D.-Y., Jiao L.-Y., Zhao G.-A. (2021). Glyoxal damages human aortic endothelial cells by perturbing the glutathione, mitochondrial membrane potential, and mitogen-activated protein kinase pathways. BMC Cardiovasc. Disord..

[2] Zhang M., Huang C., Ou J., Liu F., Ou S., Zheng J. (2024). Glyoxal in Foods: Formation, Metabolism, Health Hazards, and Its Control Strategies. J. Agric. Food Chem..

[3] Rabbani N., Thornalley P.J. (2015). Dicarbonyl stress in cell and tissue dysfunction contributing to ageing and disease. Biochem. Biophys. Res. Commun..

[4] Murata-Kamiya N., Kamiya H., Kaji H., Kasai H. (1997). Glyoxal, a major product of DNA oxidation, induces mutations at G:C sites on a shuttle vector plasmid replicated in mammalian cells. Nucleic Acids Res..

[5] Wetzels S., Wouters K., Schalkwijk C.G., Vanmierlo T., Hendriks J.J.A. (2017). Methylglyoxal-Derived Advanced Glycation Endproducts in Multiple Sclerosis. Int. J. Mol. Sci..

[6] Liu G.D., Xu C., Feng L.E., Wang F. (2015). The augmentation of O-GlcNAcylation reduces glyoxal-induced cell injury by attenuating oxidative stress in human retinal microvascular endothelial cells. Int. J. Mol. Med..

[7] Roehlecke C., Valtink M., Frenzel A., Goetze D., Knels L., Morawietz H., Funk R.H.W. (2016). Stress responses of human retinal pigment epithelial cells to glyoxal. Graefes Arch. Clin. Exp. Ophthalmol..

[8] Liu D., Chen J., Xie Y., Mei X., Xu C., Liu J., Cao X. (2022). Investigating the molecular mechanisms of glyoxal-induced cytotoxicity in human embryonic kidney cells: Insights from network toxicology and cell biology experiments. Environ. Toxicol..

[9] Knutson S.D., Sanford A.A., Swenson C.S., Korn M.M., Manuel B.A., Heemstra J.M. (2020). Thermoreversible Control of Nucleic Acid Structure and Function with Glyoxal Caging. J. Am. Chem. Soc..

[10] Shangari N., O’Brien P.J. (2004). The cytotoxic mechanism of glyoxal involves oxidative stress. Biochem. Pharmacol..

[11] Nomi Y., Aizawa H., Kurata T., Shindo K., van Nguyen C. (2009). Glutathione reacts with glyoxal at the N-terminal. Biosci. Biotechnol. Biochem..

[12] Dhananjayan K., Irrgang F., Raju R., Harman D.G., Moran C., Srikanth V., Münch G. (2019). Determination of glyoxal and methylglyoxal in serum by UHPLC coupled with fluorescence detection. Anal. Biochem..

[13] Odani H., Shinzato T., Matsumoto Y., Usami J., Maeda K. (1999). Increase in three alpha,beta-dicarbonyl compound levels in human uremic plasma: Specific in vivo determination of intermediates in advanced Maillard reaction. Biochem. Biophys. Res. Commun..

[14] Pieniazek A., Bernasinska-Slomczewska J., Gwozdzinski L. (2021). Uremic Toxins and Their Relation with Oxidative Stress Induced in Patients with CKD. Int. J. Mol. Sci..

[15] Han Y., Randell E., Vasdev S., Gill V., Gadag V., Newhook L.A., Grant M., Hagerty D. (2007). Plasma methylglyoxal and glyoxal are elevated and related to early membrane alteration in young, complication-free patients with Type 1 diabetes. Mol. Cell. Biochem..

[16] Sejersen H., Rattan S.I.S. (2009). Dicarbonyl-induced accelerated aging in vitro in human skin fibroblasts. Biogerontology.

[17] Iwata H., Ukeda H., Maruyama T., Fujino T., Sawamura M. (2004). Effect of carbonyl compounds on red blood cells deformability. Biochem. Biophys. Res. Commun..

[18] Vanholder R., van Laecke S., Glorieux G. (2008). What is new in uremic toxicity?. Pediatr. Nephrol..

[19] Sharma G.S., Bhattacharya R., Krishna S., Alomar S.Y., Alkhuriji A.F., Warepam M., Kumari K., Rahaman H., Singh L.R. (2021). Structural and Functional Characterization of Covalently Modified Proteins Formed By a Glycating Agent, Glyoxal. ACS Omega.

[20] Semchyshyn H.M. (2013). Defects in antioxidant defence enhance glyoxal toxicity in the yeast Saccharomyces cerevisiae. Ukr. Biokhim. Zh. (1999).

[21] Li X., Bakker W., Sang Y., Rietjens I.M.C.M. (2024). Absorption and intracellular accumulation of food-borne dicarbonyl precursors of advanced glycation end-product in a Caco-2 human cell transwell model. Food Chem..

[22] Gurney T.O., Oliver P.J., Sliman S.M., Yenigalla A., Eubank T.D., Nassal D.M., Miao J., Zhao J., Hund T.J., Parinandi N.L. (2022). Cardiovascular Signaling in Health and Disease: Hyperglycemic Oxoaldehyde (Glyoxal)-Induced Vascular Endothelial Cell Damage Through Oxidative Stress Is Protected by Thiol Iron Chelator, Dimercaptosuccinic Acid–Role of Iron in Diabetic Vascular Endothelial Dysfunction.

[23] Banerjee S. (2018). Glyoxal-induced modification enhances stability of hemoglobin and lowers iron-mediated oxidation reactions of the heme protein: An in vitro study. Int. J. Biol. Macromol..

[24] Kosmachevskaya O.V., Novikova N.N., Topunov A.F. (2021). Carbonyl Stress in Red Blood Cells and Hemoglobin. Antioxidants.

[25] Bonnefont-Rousselot D. (2002). Glucose and reactive oxygen species. Curr. Opin. Clin. Nutr. Metab. Care.

[26] Pieniazek A., Gwozdzinski K. (2015). Changes in the conformational state of hemoglobin in hemodialysed patients with chronic renal failure. Oxid. Med. Cell. Longev..

[27] Pieniazek A., Gwozdzinski K. (2017). Carbamylation and oxidation of proteins lead to apoptotic death of lymphocytes. Chem. Biol. Interact..

[28] Gwozdzinski K., Pieniazek A., Gwozdzinski L. (2021). Reactive Oxygen Species and Their Involvement in Red Blood Cell Damage in Chronic Kidney Disease. Oxid. Med. Cell. Longev..

[29] Lankin V.Z., Konovalova G.G., Tikhaze A.K., Shumaev K.B., Belova Kumskova E.M., Grechnikova M.A., Viigimaa M. (2016). Aldehyde inhibition of antioxidant enzymes in the blood of diabetic patients. J. Diabetes.

[30] Jabeen R., Saleemuddin M., Petersen J., Mohammad A. (2007). Inactivation and modification of superoxide dismutase by glyoxal: Prevention by antibodies. Biochimie.

[31] Khan M.A., Younus H. (2025). Superoxide Dismutase Glycation: A Contributor to Disease and Target for Prevention. Catalysts.

[32] Dodge J.T., Mitchell C., Hanahan D.J. (1963). The preparation and chemical characteristics of hemoglobin-free ghosts of human erythrocytes. Arch. Biochem. Biophys..

[33] Lowry O.H., Rosebrough N.J., Farr A.L., Randall R.J. (1951). Protein measurement with the Folin phenol reagent. J. Biol. Chem..

[34] Drabkin D.L. (1946). Spectrophotometric studies. J. Biol. Chem..

[35] Morimoto Y., Tanaka K., Iwakiri Y., Tokuhiro S., Fukushima S., Takeuchi Y. (1995). Protective effects of some neutral amino acids against hypotonic hemolysis. Biol. Pharm. Bull..

[36] Aebi H. (1984). Catalase in vitro. Methods Enzymol..

[37] Misra H.P., Fridovich I. (1972). The generation of superoxide radical during the autoxidation of hemoglobin. J. Biol. Chem..

[38] Rice-Evans C. (1991). Techniques in Free Radical Research.

[39] Yamaguchi T., Takamura H., Matoba T., Terao J. (1998). HPLC method for evaluation of the free radical-scavenging activity of foods by using 1,1-diphenyl-2-picrylhydrazyl. Biosci. Biotechnol. Biochem..

[40] Ellman G.L. (1959). Tissue sulfhydryl groups. Arch. Biochem. Biophys..

[41] Egwim I.O., Gruber H.J. (2001). Spectrophotometric measurement of mercaptans with 4,4′-dithiodipyridine. Anal. Biochem..

[42] Crowell E.A., Ough C.S., Bakalinsky A. (1985). Determination of Alpha Amino Nitrogen in Musts and Wines by TNBS Method. Am. J. Enol. Vitic..

[43] Levine R.L., Garland D., Oliver C.N., Amici A., Climent I., Lenz A.G., Ahn B.W., Shaltiel S., Stadtman E.R. (1990). Determination of carbonyl content in oxidatively modified proteins. Methods Enzymol..

